# Supplementary figures and images for: Phase-specific transcriptional patterns of the oomycete pathogen Phytophthora sojae unravel genes essential for asexual development and pathogenic processes
Source: PLoS Pathog. 2023 Mar 23;19(3):e1011256. doi: 10.1371/journal.ppat.1011256 (PMC10072465; doi:10.1371/journal.ppat.1011256)

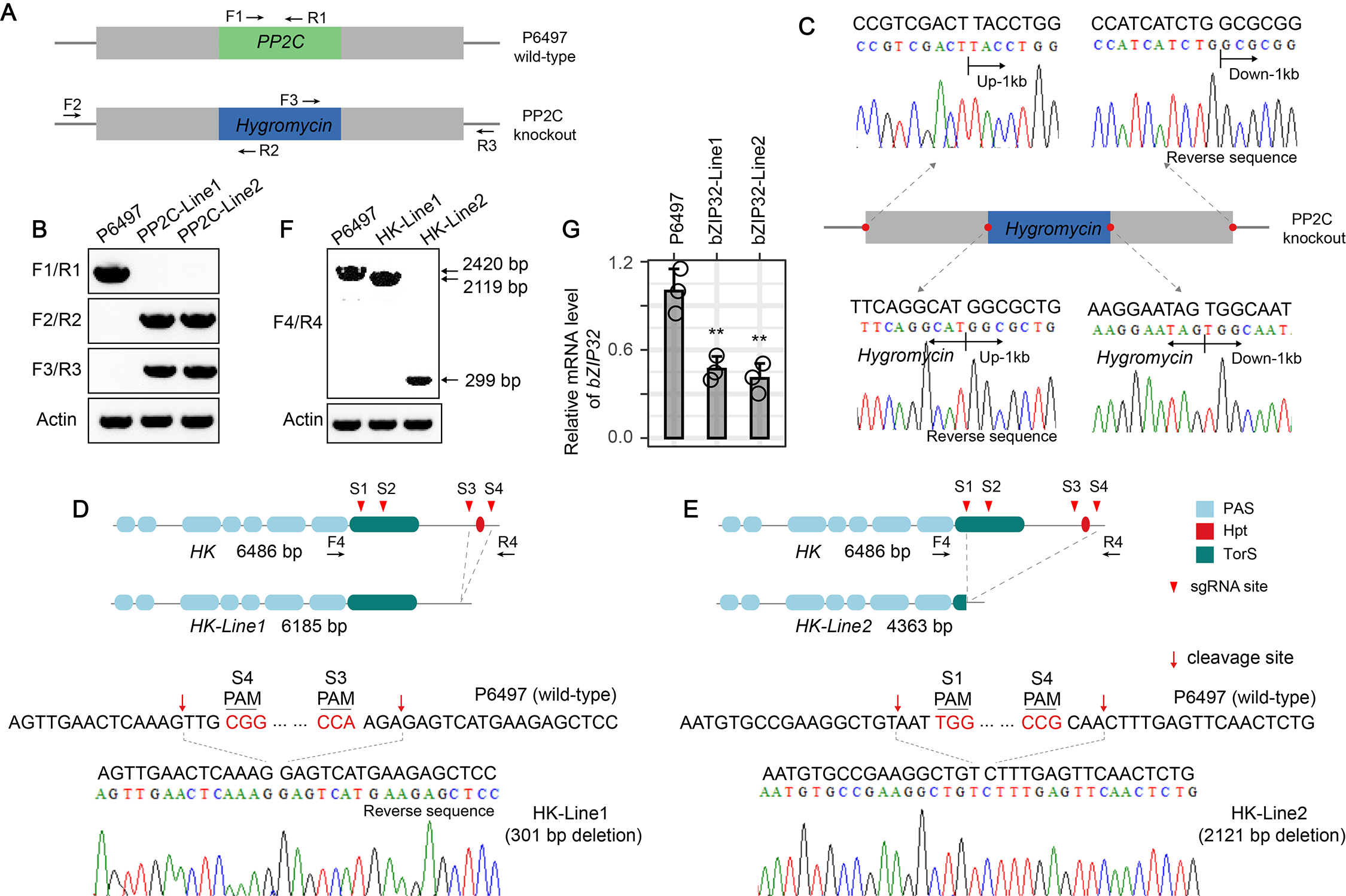

Supplement: S1 Fig — (A) Schematic diagrams for knockout strategy of PP2C. CRISPR/Cas9 mediated HDR (Homology Directed Repair) was utilized for precisely replaced of the entire gene. Also, locations of the primers used to screen the PP2C knockout mutants (F1/R1, F2/R2, and F3/R3) are indicated. (B) Analysis of genomic DNA from the wild-type (WT) and PP2C-knockout mutants (ΔPP2C-Line1 and ΔPP2C-Line2) using the primers in (A) and actin primers (positive control). (C) Sanger sequencing traces of junction regions confirming that the PP2C was precisely replaced. Red dots, junction regions. (D and E) Schematic diagrams for deletion of key regions of HK from P. sojae genome. Four sgRNAs covering key regions of HK were designed and their locations were indicated by four red triangles. Sanger sequencing traces around the deletion regions in ΔHK-Line1 (D) and ΔHK-Line2 (E). The wild-type sequence was represented above the mutated ones, with PAM (Protospacer Adjacent Motif) was indicated with red and cleavage sites was indicated with red arrows. (F) Genomic analysis of the deletion events in HK-knockout mutants (ΔHK-Line1 and ΔHK-Line2), comparing with the wild-type (WT). Primers (F4/R4) were used to covering the key region of HK. Actin gene was included as positive control. (G) The expression analysis of bZIP32 in bZIP32-Line1 and bZIP32-Line2. The bar (SD) represents the relative expression level calculated by quantitative reverse transcription PCR using the 2−ΔΔCt method. The level of gene expression in the wild-type was set equal to 1 and used to calculate the relative expression levels of the genes in the transformants. All experiments were repeated three times with similar results. **Significant difference at p < 0.01. (TIF) [file ppat.1011256.s001.tif]

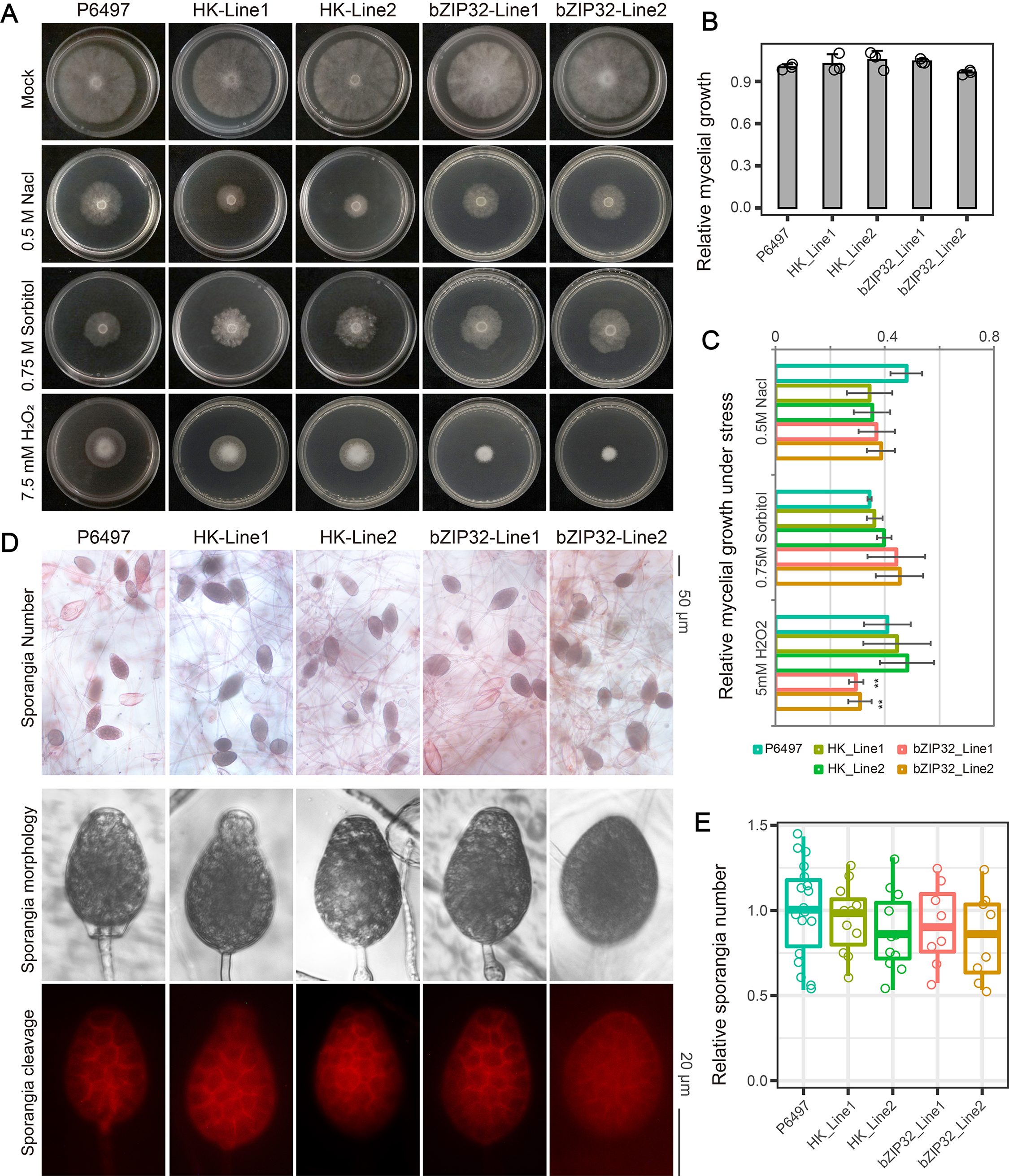

Supplement: S2 Fig — (A) Growth characteristics of the wild-type (P6497), HK and bZIP32 mutants (PP2C-Line1 and PP2C-Line2, bZIP32-Line1 and bZIP32-Line2) on V8 agar medium without (Mock) or with treatments of NaCl, Sorbitol or H2O2. (B) Relative mycelial growth of wild-type (P6497), HK and bZIP32 mutants on V8 agar medium (Mock). (C) Relative colony diameters of wild-type (P6497), HK and bZIP32 mutants after 4 days of growth in different stresses. And Relative colony diameters were calculated for each treatment relative to growth on V8 agar medium only. (D) Upper: The numbers of sporangia in wild-type (P6497), HK and bZIP32 mutants. The sporangia morphology (middle) and cytoplasm cleavage (bottom) within sporangia were observed in the wild-type (P6497), HK and bZIP32 mutants. (E) The relative numbers of sporangia in the wild-type (P6497), HK and bZIP32 mutants. (TIF) [file ppat.1011256.s002.tif]
