# Supplementary material for: Phase-specific transcriptional patterns of the oomycete pathogen Phytophthora sojae unravel genes essential for asexual development and pathogenic processes
Source: PLoS Pathog. 2023 Mar 23;19(3):e1011256. doi: 10.1371/journal.ppat.1011256 (PMC10072465; doi:10.1371/journal.ppat.1011256)
Supplement: Attachment — Submitted filename: comments.pdf [file ppat.1011256.s006.pdf]

Phase-specific transcriptional patterns of the oomycete pathogen Phytophthora sojae unravel genes essential for asexual development and pathogenic processes” by Qiu et al. is an interesting work. I have read this paper with great interest and but found it not so easy to follow. I believe the manuscript could be further improved from the suggested changes in the attached document.

Writing quality

Overall, the writing quality of the paper is poor. Proof-reading is needed. Grammar mistakes sometimes hinder the comprehension of the text.

The writing of the first subsection of the results section, “*Phytophthora sojae* exhibits greater transcriptional plasticity during developmental stages than during host infection”, was rather confusing. If the reader did not read the abstract, they would not have understood that the authors were merely trying to compare whether the “developmental stages“ model or the “infection stages“ model would be better for the analysis that followed. The way the section was written could mislead the reader to think that the authors were trying to compare between all of the ten individual stages, which would not make much sense. The way that the two models were named, especially that of the “infection stages” model was also another main source of confusion, because the different “development stages” are also present as different stages of infection. However, these names were already used in the publication of Yu et al (2011), as cited by the authors.

Other examples of poor writing quality:

Choice of vocabulary:

functional novelties. Therefore, by advancing data mining and molecular falsification,

what does molecular falsification mean???

Grammar:

highly expressed genes but these genes show similar expression patterns. In contrast,

using "but" in the main clause following the subordinate clause led by "though“

though the developmental stages had lesser genes with high expression, but they distributed in different areas (**Fig. 1B**), indicating that developmental stages exhibit more changes in gene expression patterns.

specificity than infection stages (**Fig. 1C**). These means in contrast to infection stages, germinating cyst may have a comparable amount of genes but they were specifically expressed.

amount of + non-countable nouns

These observations indicated that *P. sojae* exhibited variable mRNA profiles during the developmental stages and possessed a highly dynamic transcriptome, which referred as transcriptional plasticity.

"which" clause lacking subject

clause following "while" has no verb

clusters: M1 contains DNA replication and transcription while phosphotransferase activity in M5. Finally, the second module (M2) had the minimum 439 genes, with high

be

indicating this cellular function may ^ exclusive to mycelia and sporangia stages. As

Structuring

Good structuring of the results section, where the identification of each of the three gene and verification of their mutant phenotypes were under different subheadings.

Abstract and title reflect core substance of paper?

Yes

Graphs and table titles

Not all Figure captions are self-explanatory. The caption of Figure 2, for example, lacks information on the abbreviation of the developmental stages.

State-of-the-art

No mentioning of other transcriptomics dynamics studies on other *Phythopthora* spp. where other functional genes were identified.

Methodology and experimental design

Does the fact that the different developmental stages samples were prepared through induction and not through infection on host change anything? Could authors have compared between those induced developmental stages and developmental stages that were observed during actual infection? The “infection stages” samples were mycelium mats induced from actual plants after 1.5, 3, 6, 12 and 24h, while the MY developmental stage which were mycelium induced on 10% V8 after 48h (Yu et al, 2011), and their expression pattern do not look very similar (Figure 1B). However, in the paper by Judelsen et al (2008), similar patterns of gene expression were observed in *P. infestans* hyphae from both infected tomato and growth media.

Aim, scope and focus

The objectives that the paper accomplished was clearly included in the last paragraph of the introduction. Experiments, discussion and conclusion are sufficiently focused on the theme and main aim of the paper.

Amount and quality of data

In terms of statistics, there is no information on the scale of the “scaled expression” of

each developmental stage in each module in Figure 2. Is the first ring considered statistically ineffective? Some of the conclusions in the paper regarding these may therefore be somewhat generalized. In lines 170-171 it was mentioned that M1 was specific “primarily” to the germinating cyst (GC) stage, and then in lines 304-308, the authors completely overlooked the fact that there were the other stages in M1 as well, and just emphasized on the uniqueness of GC.

both had low expression in mycelia and sporangia. M1 was specific primarily to the germinating cyst (**Fig. 2A**), while M5 showed higher expression at the zoospore and

(**Fig. 1B and 1C**). The first module (M1), with 1553 genes, had clusters specifically and highly expressed during cyst germination (**Fig. 2A**). Gene clusters associated with DNA replication, lipase activity, and transcription were significantly enriched in this module, and these clusters did not exist in other modules (**Fig. 2A**), which also verified the uniqueness of the cyst germination stage.

Similar conclusion regarding the mycelia and sporangia stages in lines 195-201, where the GC stage also has expression in M3:

These three modules were distinguished on the high mRNA expressions, only in mycelia and sporangia (M3) or throughout the other stages (M4 and M6), suggesting their exclusive or shared functions across stages. For example, genes in M3 may participated in some biological processes that unique to mycelia and sporangia, as the maturation of sporangia. Our observation supporting this was that genes with hydrolysis-related activities appeared in M3 (**Fig. 2C**), and not in other modules, indicating this cellular function may ^be exclusive to mycelia and sporangia stages. As

Authors did not mention about the innovative aspect of their research. For example, there is literature that has previously identified HK to play an important role in infection in *P. infestans* (Xiao et al, 2019). Nevertheless, the authors verified their findings using knock-out mutants, which provides stronger support for their conclusions as compared to other studies without knock-out experiments.

References

Judelson HS, Ah-Fong AMV, Aux G, Avrova AO, Bruce C, Cakir C, da Cunha L, Grenville-Briggs L, Latijnhouwers M, Ligterink W, et al (2008) Gene

Expression Profiling During Asexual Development of the Late Blight Pathogen *Phytophthora infestans* Reveals a Highly Dynamic Transcriptome. MPMI **21**: 433–447

Xiao C, Gao J, Zhang Y, Wang Z, Zhang D, Chen Q, Ye X, Xu Y, Yang G, Yan L, et al (2019) Quantitative Proteomics of Potato Leaves Infected with Phytophthora infestans Provides Insights into Coordinated and Altered Protein Expression during Early and Late Disease Stages. IJMS **20**: 136
