# Supplementary material for: Phase-specific transcriptional patterns of the oomycete pathogen Phytophthora sojae unravel genes essential for asexual development and pathogenic processes
Source: PLoS Pathog. 2023 Mar 23;19(3):e1011256. doi: 10.1371/journal.ppat.1011256 (PMC10072465; doi:10.1371/journal.ppat.1011256)
Supplement: Attachment — Submitted filename: Response to Reviewers.docx [file ppat.1011256.s007.docx]

1/3/2023

Dear PLoS Pathogens Editorial Staff,

Thank you for handing our manuscript entitled “Phase-specific transcriptional patterns of the oomycete pathogen *Phytophthora sojae* unravel genes essential for asexual development and pathogenic processes” (#PPATHOGENS-D-22-01505). We were pleased with the positive comments from the three reviewers and appreciate their feedback that has helped us to improve this manuscript. In this revised manuscript, we added more supplementary data and modified the manuscript to address the reviewers’ concerns.

Hereby we submit the revised version of the manuscript and our point-to-point response to the comments of the reviewers. We request the same editor and reviewers as for the original submission, as our revisions closely follow the requests of those reviewers. We are looking forward to your response.

Best regards,

Yuanchao Wang

We thank the editor and the reviewers for their thoughtful and detailed input. We have done our best to address all the comments. The specific revisions that we performed in response to reviewers' comments are described below (the line number is according to the clean copy of revised manuscript).

Reviewer's Responses to Questions

**Response to Reviewer #1:**

Part I – Summary: Please use this section to discuss strengths/weaknesses of study, novelty/significance, general execution and scholarship.

Reviewer #1: Let me start by affirming that this is a very useful clustering analysis that others can use in gene discovery experiments. I very much like the organization and presentation of the figures.

However, the Gene ID numbers cited is this paper are recognizable as ones associated with earlier databases, and do not correspond (in my tests) to the gene id system of P sojae in Fungidb, where all genes are identified as Physodraft_123456. The authors need to add a row to identify equivalent genes in this database for all of their supplementary tables.
Other labs may want to use the information in Fig. 2 to characterize specific genes Therefore the Gene id information for all the clusters in Fig.2 must also be included as a supplementary table
Make a supplementary document which enables the reader to easily discern the meaning of the supplementary tables.

Response: We appreciate the reviewer's positive comments. As suggested we had uploaded a new supplementary table (**S1 Table**), which containing the Gene id information for the clusters in **Fig 2**, as well as Gene ID numbers associated with newest databases.

Part II – Major Issues: Key Experiments Required for Acceptance

Reviewer #1: no additional experiments are needed

Response: We appreciate the reviewer's positive comments.

Part III – Minor Issues: Editorial and Data Presentation Modifications
Please use this section for editorial suggestions as well as relatively minor modifications of existing data that would enhance clarity.

Reviewer #1: The use of the word “meanwhile” by the authors is in all cases inappropriate
Response: We had checked this throughout the manuscript.

Abstract
This is awkwardly phrased
Therefore, this study reboots the analysis from available transcriptome data in a model oomycete pathogen, *Phytophthora sojae*, to excavate more functional genes. A set of expression matrix of 10,953 genes across ten stages (including development and infection) were applied to recognize the transcriptional changes underlying critical stages for disease spreading.

Response: We had rephrased this sentence in revised manuscript (**Line 18-21**).

Awkard
Unlike plant diseases caused by filamentous fungi, most oomycete diseases spread
60 quickly in the fields, relying on their energetic zoospores for better colonization through
61 chemotaxis
Try
The motile zoospore stage facilitate the dispersal of these pathogens under moist or wet conditions, and(Maltese et al., 1995, Judelson & Blanco, 2005) the zoospores are able to use both general (ethanol) and host-specific signals to swim towards host roots (Maltese et al., 1995, Judelson & Blanco, 2005).
Response: Revised (**Line 69-71**).

Check:
Line 62-63: Most oomycetes produce numerous multinucleate sporangia at the end of specialized hyphae within the diseased hosts (Maltese et al., 1995).
I am assuming the authors mean on the surface of plant tissues…...
Response: Revised (**Line 72-73**).

Line 90-91: Despite such advances in oomycetes kingdom, there are still many unknowns on molecular mechanisms underlying their life cycles, especially for some critical stages in spreading of oomycete diseases.
Response: Revised (**Line 94-96**).

Line 97-98: Here, we describe the employed large-scale transcriptome profiling to analyze the dynamics of the *P. sojae* life cycle. the large-scale transcriptome profiling to analyze the dynamics of the *P. sojae* life cycle.
Response: Revised (**Line 101-102**).

Line 147-148: during the developmental stages and possessed a highly dynamic transcriptome, which referred as we describe as transcriptional plasticity.
Response: Revised (**Line 155-156**).

Awkwardly phrased
Line 151-153: To better recognize the gene expression patterns in the *P. sojae* life cycle, considering the greater transcriptional plasticity, transcript matrix of five developmental stages were further analyzed.
Perhaps…..
Next we utilized the pattern of greater transcriptional plasticity across the five developmental stages to identify novel gene expression patterns.
Response: Revised (**Line 161-162**).

Re
Line 158: As a result, six modules consisting of 7029 genes were obtained on the basis of expression clustering, which were portrayed in the radar maps in Fig. 2.
I feel this is too much like “just go look at the figure “without giving the reader a visual description of the data analysis strategy. A much better job is done in the figure legend and some of this information must be replicated in the text. Follow-up sentence(s) enabling the reader to see what was accomplished with this anlysis is clearly needed.
Response: Thanks for the comments. We had included more descriptions in revised manuscript (**Line 167-177**). Another thing here that we did not delete the first line when calculating number of genes, thus the correct number of genes was 7, 028. We had corrected this mistake in revised manuscript.

Change:
Line 169 Meanwhile, the other modules, M1 and M5, with 1553 and 605 genes, respectively…..
Line 193: Meanwhile, the fourth module (M4) was the largest among these three modules, with 2391 genes; while M3 had 1539 genes; and M6 only 501 gene.
Response: Revised. And we had checked this throughout the manuscript.

Lines 204 to 215
We noted that the M4 and M6 modules contained several genes in the serine/threonine phosphatase and serine/threonine kinase families and elected to investigate the expression patterns of these developmental regulators further.
In some cases, genes associated with the same function exhibited differences across stages in the mRNA abundance levels, probably due to the differences in the importance of these genes in molecular functions. Then, to address this issue, we performed statistical clustering of mRNAs from all stages and visual inspection of protein domains to categorize the genes into subclasses. We classified the genes from the serine/threonine phosphatase cluster into two groups, one that was relatively less expressed and the other that was upregulated at all stages of infection (Fig. 3A). We also identified multiple phosphatase domains, such as PP2A, CDC55, PP2C, HP, and DSP, in serine/threonine phosphatase-encoding genes.
Response: Revised (**Line 223-229**).

Meanwhile, IN contrast zoospores of the HK knockout mutants (HK-Line1 and HK-Line2) were not chemoattracted to root hairs. Zoospores of the wild-type strain formed cysts on and adjacent to this region of the root. While only a few cysts were observed in this region of the root in the knockout mutant strains (Fig. 4B, Root Hair).
Response: Revised (**Line 298-302**).

Change
Line 379-387: Based on the above results, the following model can be proposed (Fig. 7). We described the large-scale profiling of the *P. sojae* transcriptional changes during the transition between developmental stages. Meanwhile, we identified several candidate genes among the various stages of *P. sojae* life cycle, with significant serine/threonine phosphatase activity at the mycelial and sporangium stages, phosphotransferase activity at the zoospore and cyst stages, and transcription activity during cyst germination (Fig. 7). Subsequently, by taking advantage of genome editing techniques, the absence of these candidate genes verified their role during specific stage transitions, as well as pathogenic processes (Fig. 7).
In summary analysis of transcriptional profiling across the different developmental stages of *P sojae*. Has enabled us to functionally characterize several key developmental genes facilitating the shift of gene expression form one stage to the next (Fig. 7). These include. …..
Response: Revised (**Line 389-394**).

Awkwardly phrased.
Line 447-449: We found that, similar to fungi, *P. sojae* has a hybrid HK, this protein were integrated HK with RR domains (Fig. 4A) and involved in zoospore chemotaxis towards hosts and the subsequent cyst germination process (Fig. 4B).
Response: Revised (**Line 446-448**).

**Response to Reviewer #2:**

Part I – Summary

Reviewer #2: Aimed at identifying novel functional genes for precisely controlling oomycete diseases, this research investigated infection and developmental stage-specific gene expression in the oomycete pathogen *Phytophthora sojae*, using available transcriptome data. By performing hierarchical clustering, specification, and diversification analyses, the authors focused on the developmental stages that are more remarkable in transcriptional plasticity, leading to the genes being resolved into six distinct modules. The authors took good advantages of genome editing in *P. sojae* and generated knockout mutants for three candidate stage-specific genes.
The research generated lot of information useful for understand *P. sojae* development. However, the novelty and in-depth data analysis are not sufficient, in neither gene expression nor knockout mutants.

Response: We appreciate the reviewer's comments and suggestions. Indeed, it seems we didn’t intensively focus on neither data-analysis nor gene functional investigation. However, here we are aiming at proposing a possibility to study phase-specific genes that may play essential roles specifically in one or a few stages in *P. sojae* lifestyle by means of a combination of an explicit data-analysis pipeline and a molecular KO tool. We have to admit that there are some difficulties in both aspects such as screening phase-specific genes from limited transcriptomic samples captured 10 years ago (5 developmental stages + 5 infection time-points), and extensively verifying function for candidates constrained to the feasible but tedious CRISPR-Cas9 knockout process in *P. sojae*. By investigation of knockout mutants, we found that the three candidate genes function at specific asexual developmental stages, associated with their stage-specific expressions (**Figs 3-5**). Furthermore, we also found these candidate genes participated in infection process through multiple ways (**Figs 6 and 7**). To address the concern of the reviewer we had added additional experiments (**S2 Fig**) and new supplementary data (**S1 Table**) to further support this research.

Part II – Major Issues: Key Experiments Required for Acceptance

Reviewer #2:

1. Data compilation is significant, and too many speculations in expression data analyses.
Response: Thanks for this comment! We were realizing some description in current manuscript is not enough to clearly present the analysis results. However, cautiousness of candidate genes screening process in both pattern recognition and gene motif prediction endeavors to the reliability of results. To understand the expression data more clearly, we updated the narratives in the latest version of manuscript, please check.

2. The GO enrichment analysis described in Fig2 needs to be simplified.
Response: Thanks for this suggestion! We had simplified the GO enrichment analysis in **Fig 2**.

3. The genes up-regulated in the infection stage were mentioned many times in the manuscript, but the control was not explained.
Response: In this manuscript, the genes up-regulated in the infection stages were found by clustered heatmaps (**Fig 3A, 4A and 5A**). First of all, the expression matrix of candidate genes was normalized on the basis of a global standardization. Then a distance algorithm named Euclidean was utilized to build associations between the data points and their features. In this step we can see which points are similar or different from each other. Finally, we selected the complete-linkage as the cluster method and a dendrogram containing similarity and clustering information was generated. By this way, the candidate genes with similar expressions (high or low expression) will be clustered into a group and reflected in this dendrogram.

We realized the “upregulated” is not an accurate phrasing, and we had changed this to consistently highly expressed genes, which means it not only highly expressed in asexual developmental stages, but also in infection stages. And we added the detailed description of these clustering heatmaps in revised manuscript (**Line 490-497**).

4. In Fig3B, when supplemented with different concentrations of NaCl, sorbitol or H_2_O_2_, the colony is smaller than that of the mock. The colony of the two mutants shown in Fig3C is different after supplemented with sorbitol. In a word, the information shown in the pictures is inconsistent with the description in the manuscript. Furthermore, what is the purpose of this experiment?

Response: The **Fig 3C** shows the relative colony diameters of two mutants and the average colonies were collected from three independent replicates. Between these two mutants, though the PP2C-Line2 was little slower than PP2C-Line1 after supplemented with sorbitol, the difference was insignificant.

For the purpose of this experiment. For pathogens, their abilities in response to different stress conditions, such as osmotic stress (Bremer & Kramer, 2019, Liu *et al.*, 2021) or oxidative stress (Breitenbach *et al.*, 2015, Yaakoub *et al.*, 2022), were important indicators for their performances during plant-pathogens interactions. On the basis of these experiment, we found that the PP2C mutants more insensitive to H_2_O_2_ treatments and next conduct DAB straining (**Fig 7C**) was conduct on their infectious hyphae. And we found the PP2C mutants show much more accumulation of peroxide.

5. The reasons for selecting the three genes representing different development stages need to be explained more fully. Since genes of different development stages were focused as stated, why selected PP2C, HK and bZIP32 that were up-regulated during infection? Furthermore, knockout mutants of these genes were not examined for their roles in plant infection, given that they were up-regulated during infection. In addition, high expression or up-regulated expression need more detailed description.
Response: we realized that the “up-regulated” here will make readers confused and we had changed this to consistently highly expressed genes. Also, we added more detailed description of genes with high expressions to the Materials and Methods in revised manuscript (**Line 490-497**). The reasons that we selected the three genes were that they not only highly expressed in asexual developmental stages, but also in infection stages. Furthermore, the roles of PP2C, HK and bZIP32 in plant infection were contained in **Fig 6**. First of all, the overall virulence assay of all the mutants were conduct on soybean, the pathogen’s specific host plant. They all exhibit significantly decreased virulence on soybeans **(Fig 6A)**. Furthermore, to explore the reason for the impeded virulence of these mutants, epidermal cells from the seedling inoculation sites were excised at different time points and observed. All mutants displayed significantly reduced infectious ostioles **(Fig 6E** in revised manuscript**)**. And the PP2C and bZIP32 mutants induced severe cell necrosis around the infection sites. Their performances during infection stages were also matched with their phenotypes in developmental stages.

We realized the “upregulated” is not an accurate phrasing, and we had changed this to consistently highly expressed genes, which means it not only highly expressed in asexual developmental stages, but also in infection stages. And we added the detailed description of these clustering heatmaps in revised manuscript (**Line 490-497**).

6. Silencing efficiencies of target genes in all mutants are not provided.
Response: We had added the silencing efficiencies of targets in *bZIP32* silencing mutants and it was added as **Fig S1G** in revised manuscript.

For the PP2C and HK mutants, they were generated by CRISPR/Cas9 mediated gene editing system. The knockout mutants can be confirmed by verifying where the target DNA sequences are deleted or edited in the genome. To this end, genomic DNA from all the mutants were extracted and following genomic PCR were conduct to verify whether the target DNA sequence were edited or knockout.

For *PP2C* mutants, CRISPR/Cas9 mediated HDR (Homology Directed Repair) strategy was utilized, in which the target *PP2C* ORF was precisely replaced by the *hph* (hygromycin B phosphotransferase) ORF. Thus, two rounds of primers were used to check these HDR mutants (**S1A Fig**). The first round primers were located inside the *PP2C* ORF to detect deletion events. As we can see in **S1B Fig (F1/R1)**, the *PP2C* knockout mutants showed no bands of *PP2C* sequence, indicating that deletion events may have occurred. The second round of PCRs used primers located outside the *PP2C* homology arms (to avoid the interference of transferred plasmids) and within the *hph* gene to detect homologous recombination events. As we can see in **S1B Fig (F2/R2, F2 /R3)**, the *PP2C* knockout mutants showed clear bands of replaced sequences, indicating that the original *PP2C* ORF in the *P. sojae* genome had been replaced by the *hph* ORF. Furthermore, Sanger sequencing across the junctions of the flanking sequences and *hph* was also consistent with the replacement of the *PP2C* ORF with the *hph* gene **(S1C Fig)**.

For *HK* mutants, because of the long stretches of DNA sequence of *HK* gene (full-length *HK* ORF is 6486 bp), CRISPR/Cas9 mediated NHEJ (non-homologous end-joining) strategy was utilized, in which the direct deletions were generated in the *HK* ORF. In this strategy, only sgRNA plasmids were used and the primers were located outside the sgRNA target points. As in **S1E Fig**, HK mutants show clear smaller bands comparing to wild-type (P6497), indicating that the direct deletion had generated in *HK* ORF. The following sanger sequencing confirmed their deletions in *HK* ORF.

We recognize that the description of these mutants is not very clear. We had included more details of the verifications of mutants in the part of Materials and Methods (**Line 517-541**).

7. According to the description in the manuscript, the genes in M4 and M6 were highly expressed not only in mycelia and sporangia stages, but also in the other three development stages. The author finally took the genes encoding serine/threonine phosphatases in M4 as candidates and selected PP2C as the target gene. Although PP2C was proved to affect colony morphology and the number of sporangia, how is its role in other development stages?
Response: As shown in **Fig 2D** and **Fig 3A**, the PP2C was highly expressed in mycelia, sporangia, and germinating cyst stages. The knockout of PP2C gene not only exhibit significant reduction (87%–92%) in sporangium number, but also abnormal in sporangial cytoplasm cleavage. These effects mean the PP2C mutants have no other developmental stages, including zoospore, cyst, and germinating cyst stages. Thus, we cannot observe the role of PP2C in other developmental stages.

8. HK-line2 is not available in Fig6A, statistical analysis on lesion area is also needed.
Response: Thanks for point out this, HK-line2 was marked in Fig. 6A in revised Figures. Also, the statistical analysis on lesion area was added to **Fig 6B** and mentioned it in revised manuscript (**Line 362 and 367**).

9. How is the phenotype change of HK and bZIP32 mutants in the development stages of mycelia and sporangia.
Response: We had included the phenotype change of HK and bZIP32 mutants in mycelia and sporangia stages in **S2 Fig** and in revised manuscript (**Line 420-423**).

10. The model in Figure7 is poorly proposed, a simple model is suggested.

Response: We had provided a new model in revised manuscript, which is simpler than the old one, please check that in newly uploaded **Fig 7**.

Part III – Minor Issues: Editorial and Data Presentation Modifications
Please use this section for editorial suggestions as well as relatively minor modifications of existing data that would enhance clarity.

Reviewer #2: a. Fig S1 is not marked with E.
Response: Added.

b. Line138-139: SP and MY should be marked.
Response: Revised.

c. Line189: M8?
Response: Revised.

d. Line282: he?

Response: Revised.

**Response to Reviewer #3:**

Phase-specific transcriptional patterns of the oomycete pathogen *Phytophthora sojae* unravel genes essential for asexual development and pathogenic processes” by Qiu et al. is an interesting work. I have read this paper with great interest and but found it not so easy to follow. I believe the manuscript could be further improved from the suggested changes in the attached document.

Response: We thank the reviewer for the positive comments. As suggested changes in the attached document, we had revised corresponding parts in revised manuscript, please check.

**Writing quality:** Overall, the writing quality of the paper is poor. Proof-reading is needed. Grammar mistakes sometimes hinder the comprehension of the text.

Response: We appreciate the reviewer's suggestions. All the writing mistakes pointed by reviewers had been revised. In addition, we had corrected the grammar mistakes throughout the manuscript.

The writing of the first subsection of the results section, “*Phytophthora sojae* exhibits greater transcriptional plasticity during developmental stages than during host infection”, was rather confusing. If the reader did not read the abstract, they would not have understood that the authors were merely trying to compare whether the “developmental stages” model or the “infection stages” model would be better for the analysis that followed. The way the section was written could mislead the reader to think that the authors were trying to compare between all of the ten individual stages, which would not make much sense. The way that the two models were named, especially that of the “infection stages” model was also another main source of confusion, because the different “development stages” are also present as different stages of infection. However, these names were already used in the publication of Yu et al (2011), as cited by the authors.

Response: Thanks for these suggestions and concerns! For clearly distinguish the “development stages” and “infection stages”, we had changed the “developmental stages” to “asexual stages”. And we also rechecked this throughout the manuscript for more accurate statement.

**Structuring:** Good structuring of the results section, where the identification of each of the three gene and verification of their mutant phenotypes were under different subheadings.

Abstract and title reflect core substance of paper? Yes

Response: We appreciate the reviewer's positive comments.

**Graphs and table titles:** Not all Figure captions are self-explanatory. The caption of Figure 2, for example, lacks information on the abbreviation of the developmental stages.

Response: Revised (**Line 795-796**). And we also rechecked this throughout all the Figure captions.

**State-of-the-art:** No mentioning of other transcriptomics dynamics studies on other *Phythopthora* spp. where other functional genes were identified.

Response: Thanks for these suggestions! We had added this part in revised manuscript (**Line 450-453**).

**Methodology and experimental design:** Does the fact that the different developmental stages samples were prepared through induction and not through infection on host change anything? Could authors have compared between those induced developmental stages and developmental stages that were observed during actual infection? The “infection stages” samples were mycelium mats induced from actual plants after 1.5, 3, 6, 12 and 24h, while the MY developmental stage which were mycelium induced on 10% V8 after 48h (Yu et al, 2011), and their expression pattern do not look very similar (Figure 1B). However, in the paper by Judelsen et al (2008), similar patterns of gene expression were observed in *P. infestans* hyphae from both infected tomato and growth media.

Response: This is a technical issue. It is due to the fact that infection process is complex and asynchronous. For example, in given infected tissues caused by oomycete pathogens, the developments of the pathogens are asynchronous in the overall context of total tissues and it is very hard to isolate the pure samples from infected tissues that can represent particular developmental stages. Thus, it is a good strategy to prepare different developmental stages samples through induction.

The similar or not similar expression patterns between hyphae from infected and growth media may be caused by the numbers of detected genes and sampling times. In the paper by Judelsen (Judelson *et al.*, 2008), it contains only one sampling time was collected during infection (at 4-day infected tomato) and five genes were displayed to have similar patterns. However, in this study, after filtering the low abundance genes (total counts less than 10), 10,953 genes were analyzed and displayed in Figure 1B. Besides, Multiple sampling times were contained (From 1.5 to 24 hpi). In terms of whole transcriptome, it is reasonable that genes show similar or not similar patterns between mycelia and infection stages. These two stages have similar biological function, such as nutrient uptake. Also, it is not surprising that genes show different expression patterns, for example infection stages needs many enzymes to attack host for better colonization. When we included multiple infection time points, it is bound to further increase the difference between mycelia and stages.

**Aim, scope and focus:** The objectives that the paper accomplished was clearly included in the last paragraph of the introduction. Experiments, discussion and conclusion are sufficiently focused on the theme and main aim of the paper.

Response: We appreciate the reviewer's positive comments.

**Amount and quality of data:** In terms of statistics, there is no information on the scale of the “scaled expression” of each developmental stage in each module in Figure 2. Is the first ring considered statistically ineffective? Some of the conclusions in the paper regarding these may therefore be somewhat generalized. In lines 170-171 it was mentioned that M1 was specific “primarily” to the germinating cyst (GC) stage, and then in lines 304-308, the authors completely overlooked the fact that there were the other stages in M1 as well, and just emphasized on the uniqueness of GC.

Similar conclusion regarding the mycelia and sporangia stages in lines 195-201, where the GC stage also has expression in M3:

Response: Thanks for these suggestions and concerns! Regarding to statistics in pattern recognition, Short Time-series Expression Miner (STEM) was conducted followed default settings. Before we load the expression matrix into STEM, data was log2 transformed. In standard STEM pipeline, scaled method “Normalize data” was conducted transforming the vector to (0, v1 − v0, v2 − v0, ..., vn − v0), in which v0 is SP. We made some changes in order to clearly delineate the results that confused reviewers in current manuscript, please see **Line 479-489**. In terms of phase-specific pattern, for instance, genes in M1 dominantly express in GC stage, literally, which doesn’t mean the expression level is 0 in the rest four stages. Indeed, we practically only focus on the stage of GC to functionally disentangle the meaning of such genes keep relative high level in GC but low level in the rest by testing phenotypes intimately associated with GC. However, it’s hard to tell these genes don’t play roles at all in the rest stages. In the same way, genes dominantly expressed in one to a few stages were the candidates for the further analysis.

Authors did not mention about the innovative aspect of their research. For example, there is literature that has previously identified HK to play an important role in infection in *P. infestans* (Xiao et al, 2019). Nevertheless, the authors verified their findings using knock-out mutants, which provides stronger support for their conclusions as compared to other studies without knock-out experiments.

Response: Thanks for the suggestions. We added the innovative aspect of this research to the part of Discussion in revised manuscript **(Line 448-450)**.

Reference:

Breitenbach M, Weber M, Rinnerthaler M*, et al.*, 2015. Oxidative stress in fungi: its function in signal transduction, interaction with plant hosts, and lignocellulose degradation. *Biomolecules* **5**, 318-42.

Bremer E, Kramer R, 2019. Responses of Microorganisms to Osmotic Stress. *Annual review of microbiology* **73**, 313-34.

Judelson HS, Ah-Fong AM, Aux G*, et al.*, 2008. Gene expression profiling during asexual development of the late blight pathogen *Phytophthora infestans* reveals a highly dynamic transcriptome. *Molecular Plant-Microbe Interactions* **21**, 433-47.

Liu Y, Gong X, Li M*, et al.*, 2021. Effect of Osmotic Stress on the Growth, Development and Pathogenicity of Setosphaeria turcica. *Front Microbiol* **12**, 706349.

Yaakoub H, Mina S, Calenda A*, et al.*, 2022. Oxidative stress response pathways in fungi. *Cellular and Molecular Life Sciences* **79**, 333.
